# Epidemiological, Clinical and Histological Characteristics of HBV/HDV Co-Infection: A Retrospective Cross-Sectional Study in Guangdong, China

**DOI:** 10.1371/journal.pone.0115888

**Published:** 2014-12-22

**Authors:** Baolin Liao, Fuchun Zhang, Siwei Lin, Haolan He, Yu Liu, Jiansheng Zhang, Ying Xu, Junqing Yi, Yunqing Chen, Huiyuan Liu, Zhanhui Wang, Weiping Cai

**Affiliations:** 1 Department of Infectious Disease, Guangzhou No. 8 People's Hospital, Guangzhou Medical University, Guangzhou, China; 2 Department of Third Internal Medicine, Yuexiu District Traditional Chinese Medicine Hospital, Guangzhou, China; 3 Hepatology Unit and Department of Infectious Diseases, Nanfang Hospital, Southern Medical University, Guangzhou, China; University of Pisa, Italy

## Abstract

**Background:**

The epidemiology of hepatitis D virus (HDV) in China is fairly unknown. The mechanisms whereby HDV leads to accelerated liver disease in hepatitis B virus (HBV)/HDV co-infected patients and the histological characteristics of chronic hepatitis D (CHD) patients need further investigation.

**Methods:**

The prevalence of HDV was retrospectively evaluated in all consecutive hospitalized patients with chronic HBV infection from May 2005 to October 2011. HBV/HDV co-infected patients and HBV mono-infected patients were compared clinically and histologically. Significant histological abnormality was defined as significant necroinflammation (grade ≥A2) and/or significant fibrosis (stage ≥ F2).

**Results:**

6.5% of patients (426/6604) tested positive for IgM anti-HDV. HDV was more common in patients over 50 years old than those under 50 (11.7% vs. 5.1%, P<0.001). HBV/HDV co-infected patients had higher frequencies of end-stage liver disease (ESLD) than HBV mono-infected patients, and HDV co-infection was an independent risk factor for ESLD (OR: 1.428, 95%CI: 1.116–1.827; P = 0.005). The HBV DNA levels in the HBV/HDV group were significantly lower than the HBV group in chronic hepatitis patients (median: 6.50 log_10_copies/mL vs 6.80 log_10_copies/mL, P = 0.003), but higher than the HBV group in ESLD patients (median: 5.73 log_10_copies/mL vs 5.16 log_10_copies/mL, P<0.001). When stratified by alanine aminotransferase (ALT) level, 46.7%, 56.5% and 80.5% of CHD patients had significant necroinflammation and 86.7%, 87.0% and 90.3% had significant fibrosis with ALT 1–2×upper limit normal (ULN), 2–5×ULN and>5×ULN respectively.

**Conclusion:**

The prevalence of HDV is not low in patients with chronic HBV infection. HDV may contribute to progression to ESLD through late-phase HBV DNA reactivation.

## Introduction

Hepatitis D virus (HDV) was first discovered by Mario Rizzetto in 1977 that can only propagate in patients with hepatitis B virus (HBV) [1]. It is estimated that more than 18 million have evidence of exposure to HDV among the 350 million chronic carriers of HBV worldwide [2]. HDV was traditionally highly endemic in Mediterranean countries [3], but its prevalence has declined significantly in many regions primarily due to vaccination efforts against HBV in the last few decades. For instance, the prevalence of HDV infection in HBsAg chronic carriers decreased from 24% in 1990 to 8.5% in 2006 in Italy [4]. As such, HDV is considered “a vanishing disease” in Europe [5]–[8]. However, rates of infection have plateaued in Germany and Italy [9], [10], and may even be increasing in the United Kingdom in recent years [11]. Hence, more studies are needed on the epidemiology of HDV worldwide [12].

China has one of the largest HBV infected populations in the world, but thus far, no nationwide study has been undertaken to evaluate the epidemiology of HDV. This knowledge gap may mask a major public health concern. Another chief consideration regarding HBV/HDV co-infected patients is the higher risk of progression to liver cirrhosis (LC) and hepatocellular carcinoma (HCC) compared to HBV mono-infected patients [13]–[17]. A 28-year prospective cohort study has shown an annual cirrhosis rate of 4% and HCC rate of 2.8% in patients with persistent HDV co-infection, which is higher than HBV mono-infected patients, and that HDV replication is an independent predictor for liver-related mortality [18]. However, another study reported similar frequencies of HCC between HBV/HDV co-infected and HBV mono-infected patients [11]. The mechanism by which HDV hastens the progression to end-stage liver disease (ESLD) has not been clearly demonstrated and correctly assessing histological abnormalities within the liver is very important in evaluating disease severity and management. Although transient elastography is useful for detecting advanced hepatic fibrosis in chronic hepatitis B (CHB) and chronic hepatitis C patients, it has not been shown to be accurate for chronic hepatitis D (CHD) patients and liver biopsy remains the gold standard for assessing degree of fibrosis [19], [20]. There have been few studies to date investigating the histological characteristics of CHD patients.

The aim of this study is to determine the prevalence of HBV/HDV co-infection in the Guangdong province. We then investigated clinical and histological differences between HBV/HDV co-infection and HBV mono-infection, with a focus on identifying risk factors for progression to ESLD.

## Methods

### Patients

The study protocol was conducted within the guidelines of the 1975 Declaration of Helsinki, and was approved by the ethics committee of Guangzhou No. 8 People's hospital. Due to the retrospective nature of the study, written informed consent could not be obtained from all patients. All data was anonymized and de-identified prior to analysis.

A three-step process for analyzing HBV/HDV co-infected patients was performed. First we evaluated the prevalence of HDV in the Guangdong province by screening all consecutive patients in Guangzhou No. 8 People's Hospital from May 2005 to October 2011. The inclusion criteria were the following: (1) HBsAg positive for at least the previous 6 months, (2) testing performed for HAV, HCV, HDV, HEV and HIV antibodies, and (3) not received any antiviral therapies. HBV/HDV co-infected patients were defined as having positive serum IgM antibody to HDV (IgM anti-HDV) for at least 3 months [21].

In the second step, we compared HBV/HDV co-infected patients and HBV mono-infected patients to explore risk factors associated with ESLD. The inclusion criteria were the same as above. The exclusion criteria were the following: (1) HAV, HCV, HEV or HIV co-infection or triple-infection, (2) use of hepatotoxic drugs, (3) regular alcohol consumption (>20 grams per day for females or>40 grams per day for males) and (4) patients with acute on chronic liver failure (ACLF). ESLD was defined as patients with liver cirrhosis (LC) and/or hepatocellular carcinoma (HCC). The presence of LC was defined histologically, clinically or biochemically: (1) histological findings consistent with cirrhosis, (2) LC was clinically assessed when the patients presented with oesophageal varices or had an episode of ascites, gastrointestinal bleeding or hepatic encephalopathy in their past medical history and (3) a biochemical–ultrasonographic diagnosis was made when at least two of the following features coexisted: platelet count below 100×10^9^/L, aspartate aminotransferase (AST)/alanine aminotransferase (ALT) ratio>1, cholinesterase below the lower limit of normal, international normalized ratio (INR)>1.5 and/or splenomegaly (spleen size>12 cm) [22]. HCC was diagnosed based on the following criteria: a histopathological examination, a positive lesion detected by at least 2 different imaging techniques (abdominal ultrasonography, angiogram, or computed tomography), or by 1 imaging technique and a serum α-fetoprotein level of 400 ng/mL or greater. All the enrolled patients were then categorized into two subgroups clinically: chronic hepatitis (early-phase) and ESLD (late-phase).

In the third step, we investigated the liver histological characteristics in CHD patients. Patients were biopsied if they voluntarily agreed to full assessment of the severity of liver fibrosis and inflammation and receive further treatment advice after biopsy. Seventy-nine CHD patients had liver biopsy in all included HBV/HDV co-infected patients, and they were compared to 240 treatment naive CHB patients from the same cohort we recruited and published previously [23].

### Biochemical and serological test

Biochemical tests and complete blood cell counts were performed using routine automated analyzers. IgM anti-HDV was detected by enzyme-linked immunosorbent assay kit (Zhongshan Biotech Co, China). HBV and other markers (HAV, HCV, HEV and HIV) were detected by chemiluminescent enzyme immunoassay (Abbott Laboratories, Chicago, IL, USA). Serum ALT, AST, and prothrombin activity (PTA) levels were determined by commercial kits. The upper limit of normal (ULN) of serum ALT and AST was 40U/L for both males and females. Serum level of HBV DNA was measured by real-time PCR with a lower detection limit of 1000 copies/mL (DaAn Gene Co, China).

### Liver biopsy and histology assessment

Liver biopsies were obtained using a 16G core aspiration needle and considered adequate sampling with a biopsy length of at least 1.5 cm and six or more portal tracts. Only pre-treatment biopsies were included. Biopsies were fixed, paraffin-embedded, and stained with hematoxylin and eosin for morphological evaluation and Masson's trichrome stain for assessment of fibrosis. The pathologist reviewing biopsy specimens was blinded to the biochemical and virologic results of the patients. Liver biopsies were scored using the Metavir scoring system for both inflammation grade and fibrosis stage [24]. Significant histological abnormality was defined as significant necroinflammation (grade ≥A2) and/or significant fibrosis (stage ≥F2).

### Statistical analysis

Data was analyzed using the statistical package SPSS (version13.0; SPSS, Inc., Chicago, IL). Results were presented as a median (10–90% percentile) or number (%) of patients. Levels of HBV DNA were expressed as log_10_copies/mL. Chisquare was used for categorical variables. The Mann-Whitney test was used for similar comparison of nonparametric data. A univariate analysis was first performed to determine if any variables were associated with ESLD and significant histological abnormalities. Multivariate logistic regression was then used to determine whether the identified variables from above were independent risk factors associated with ESLD and significant histological abnormalities. A two tailed P-value of <0.05 was considered statistically significant.

## Results

### Epidemiology of HDV

**Figure 1:**
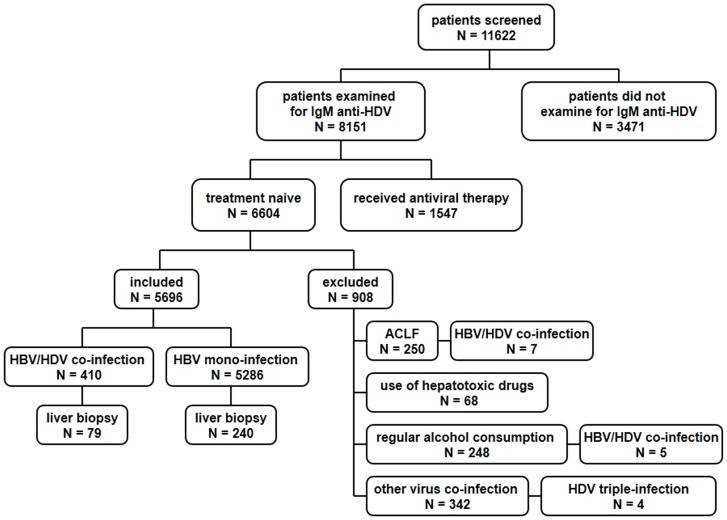
Flow chart of study design. First, 11622 consecutive patients were screened from May 2005 to October 2011. Only 6604 treatment naive patients with chronic HBV infection were tested for IgM anti-HDV and the prevalence of HDV was investigated. Second, patients with ACLF, use of hepatotoxic drugs, regular alcohol consumption and other virus co-infection or triple-infection were excluded. The clinical characteristics of the remaining patients were then compared by HDV status. Finally, liver biopsies of 79 CHD patients were compared to those of 240 CHB patients.

**Figure 2:**
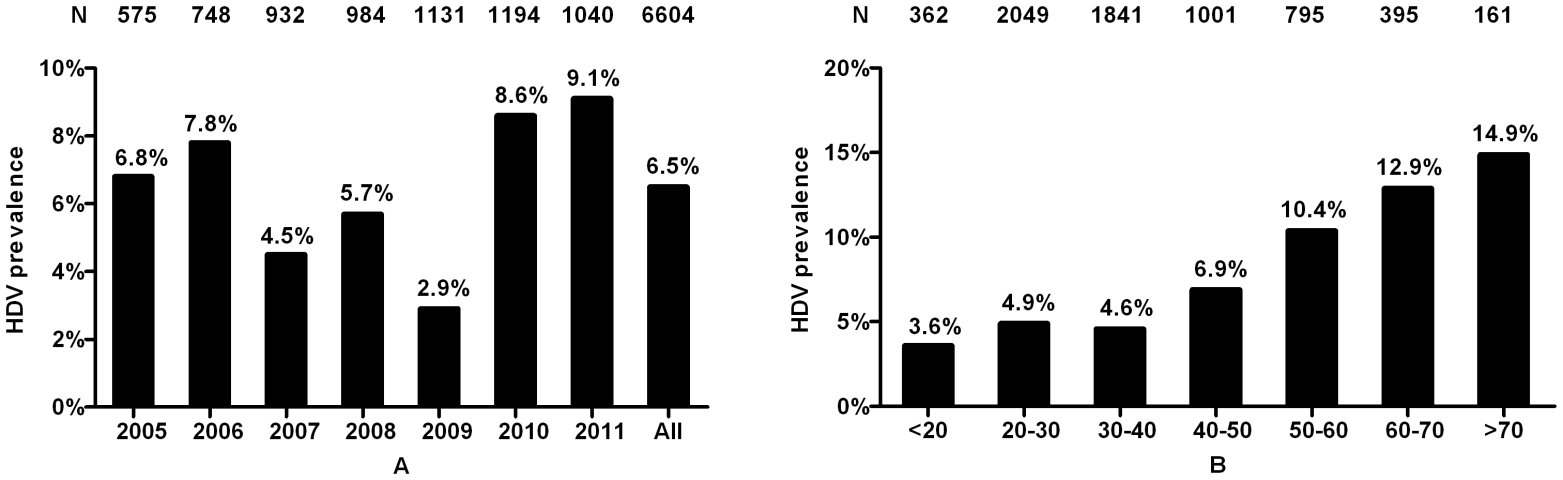
Distribution of HDV stratified by years and age. (A) Distribution of HDV stratified by years. The prevalence of HDV declined from 6.8% in 2005 to 2.9% in 2009, but then increased to 9.1% in 2011. The prevalence of HDV was 6.5% among all patients. (B) Distribution of HDV stratified by age. The prevalence of HDV correlated with age, with the lowest (3.6%) in patients under 20 years old and the highest (14.9%) in patients over 70 years old. Patients over 50 years old have higher frequencies of HDV compared to those under 50 (11.7% vs. 5.1%, P<0.001).

The algorithm of sample selection on HBV/HDV co-infection in this study is shown in Fig. 1. Out of 11622 cases screened from May 2005 to October 2011, 29.9% (3471/11622) of patients did not receive IgM anti-HDV testing. In the remaining 8151 patients tested for IgM anti-HDV, 6604 were treatment naive. Among them, 6.5% (426/6604) of patients were serum IgM anti-HDV positive including 7 patients with ACLF, 5 patients with regular alcohol consumption and 4 patients with triple virus infections. The prevalence of HDV was lowest in 2009 and the highest in 2011 (Fig. 2A) and showed an increasing trend with age (Fig. 2B). Patients over 50 years old had much higher frequencies of HDV co-infection compared to those under 50 (11.7% vs. 5.1%, P<0.001).

### Clinical characteristics of HBV/HDV co-infected patients

We analyzed clinical characteristics of 5696 patients divided by HDV status. Demographic profiles and clinical parameters from both groups are shown in Table 1. Results indicated male gender, older age and HDV co-infection were associated with ESLD by univariate analysis (all P<0.001). Results of multivariate analysis demonstrated male gender (odds ratio [OR]: 2.332, 95% confidence interval [CI]: 1.961–2.772; P<0.001), age over 50 (OR: 15.386, 95%CI: 13.015–18.188; P<0.001) and HDV co-infection (OR: 1.428, 95%CI: 1.116–1.827; P = 0.005) were independently associated with ESLD.

**Table 1:** Demographic and clinical characteristics of HBV/HDV co-infected patients.

	HBV/HDV (N = 410)	HBV (N = 5286)	*P* value
Age (years)	41 (23–65)	33 (21–57)	0.005
Sex-male (n, %)	278 (67.8%)	4042 (76.5%)	<0.001
HBeAg positive (n, %)	165 (40.2%)	3047 (57.6%)	<0.001
PTA (%)	77.42 (42.11–109.09)	85.71 (46.98–120.00)	<0.001
PLT (×10^9^/L)	140 (59–229)	171 (76–252)	<0.001
ALT (U/L)	152 (43–543)	109 (36–637)	<0.001
AST (U/L)	145 (53–427)	88 (33–386)	<0.001
HBV DNA (log_10_copies/mL)	6.11 (3.97–7.72)	6.39 (3.62–7.97)	0.001
ESLD (n, %)	184 (44.9%)	1492 (28.2%)	<0.001

Parameters are expressed as median (10–90% percentile) or number (%)

ESLD, end-stage liver disease; PTA, prothrombin activity; PLT, platelet; ALT, alanine aminotransaferase; AST, aspirate aminotransferase;

### Characteristics of HBV/HDV co-infected patients with ESLD

We then performed subgroup analysis on HBV/HDV co-infected patients clinically. The demographic profiles and parameters from both groups are shown in Table 2. Among chronic hepatitis patients, HBV/HDV co-infected patients were less likely to have positive HBeAg and had lower HBV DNA levels compared to HBV mono-infected patients. However, in ESLD patients, HBV/HDV co-infected patients had a similar age and HBeAg status to patients with HBV mono-infection. Moreover, the median level of HBV DNA was 5.73log_10_ copies/mL in HBV/HDV co-infected patients compared to 5.16log_10_ copies/mL in patients with HBV mono-infection (P<0.001).

**Table 2:** Characteristics of HBV/HDV co-infected patients with chronic hepatitis and ESLD.

	Chronic hepatitis			ESLD		
	HBV/HDV (N = 226)	HBV (N = 3794)	*P* value	HBV/HDV (N = 184)	HBV (N = 1492)	*P* value
Age (years)	31 (22–55)	33 (24–48)	NS	50 (34–65)	50 (33–67)	NS
Sex-male (n, %)	143 (63.3%)	2829 (74.6%)	<0.001	135 (73.4%)	1213 (81.3%)	0.011
HBeAg positive (n, %)	132 (58.4%)	2730 (72.0%)	<0.001	33 (17.9%)	317 (21.2%)	NS
PTA (%)	87.27 (66.29–117.04)	92.31 (63.16–123.08)	0.003	55.81 (31.93–93.76)	62.34 (32.00–97.96)	NS
PLT (×10^9^/L)	164 (106–244)	186 (122–257)	<0.001	84 (44–164)	103 (46–213)	0.001
ALT (U/L)	239 (66–704)	143 (43–723)	<0.001	79 (35–253)	58 (29–315)	<0.001
AST (U/L)	171 (52–451)	89 (32–399)	<0.001	112 (52–420)	83 (41–346)	<0.001
HBV DNA (log_10_copies/mL)	6.50 (3.95–7.91)	6.80 (4.18–8.08)	0.003	5.73 (3.98–7.32)	5.16 (3.11–7.12)	<0.001

Parameters are expressed as median (10–90% percentile) or number (%)

ESLD, end-stage liver disease; PTA, prothrombin activity; PLT, platelet; ALT, alanine aminotransaferase; AST, aspirate aminotransferase; NS, not significant

### Liver histological characteristics of CHD patients

**Figure 3:**
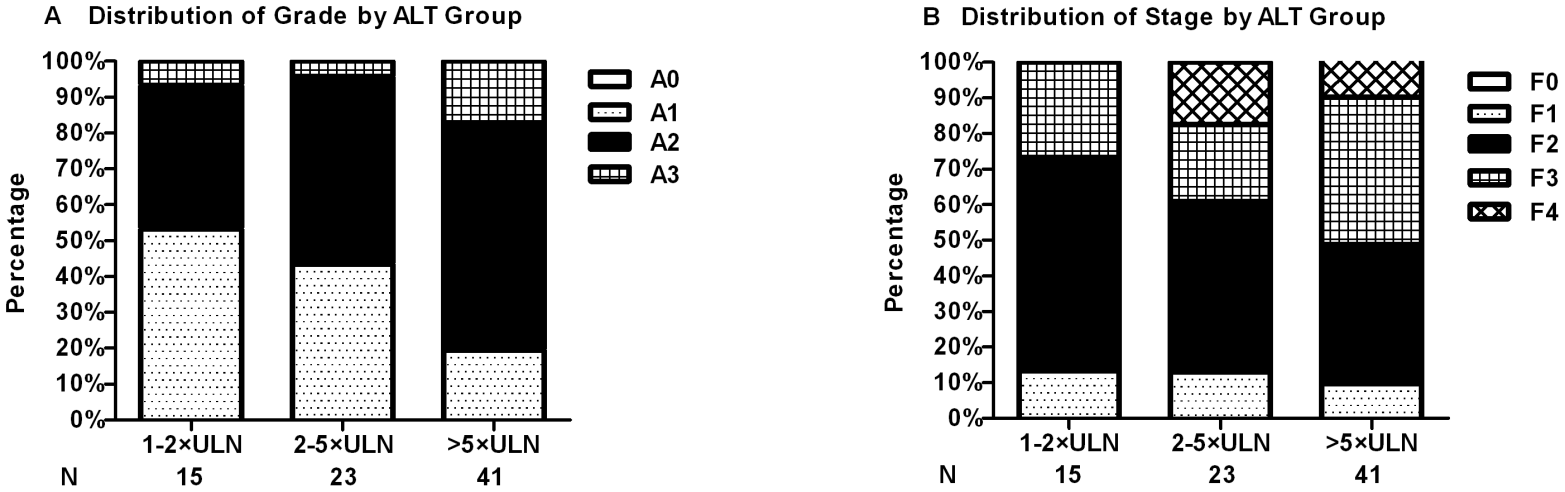
Necroinflammation grade and fibrosis stage in CHD patients. (A) Necroinflammation grade in CHD patients. Significant necroinflammation (≥A2) was found 46.7%, 56.5% and 80.5% in ALT 1–2×ULN, 2–5×ULN and>5×ULN groups, respectively. Significant necroinflammation in>5×ULN group were much higher than those in the 1–2×ULN and 2–5×ULN group (both P<0.05). (B) Fibrosis stage in CHD patients. Significant fibrosis (≥F2) was found 86.7%, 87.0% and 90.3% in ALT 1–2×ULN, 2–5×ULN and>5×ULN groups, respectively. Significant fibrosis in>5×ULN group were similar with those in 1–2×ULN and 2–5×ULN group (both P>0.05).

Seventy-nine CHD patients underwent liver biopsy. Results were compared to liver biopsy from 240 CHB patients. Although there were no differences in age, sex, HBeAg status and levels of HBV DNA between CHD and CHB patients, CHD patients had higher levels of ALT and AST as well as lower levels of PLT count and PTA compared to CHB patients (Table 3). Furthermore, CHD patients had higher frequencies of significant necroinflammation (67.1% vs. 43.8%, P<0.001) and significant fibrosis (88.6% vs. 78.3%, P = 0.044) compared to CHB patients. When stratified by ALT level, 46.7%, 56.5% and 80.5% had significant necroinflammation, and 86.7%, 87.0% and 90.3% had significant fibrosis in CHD patients with ALT 1–2×upper limit normal (ULN), 2–5×ULN and>5×ULN respectively (Fig. 3). The frequency of significant necroinflammation in the>5×ULN group was much higher than those in the 1–2×ULN and the 2–5×ULN group (both P<0.05), while significant fibrosis was similar among all three groups.

**Table 3:** Characteristics of CHD patients with liver biopsy.

	CHD (N = 79)	CHB (N = 240)	*P* value
Age	28 (22–43)	28 (20–41)	NS
Sex-male (n, %)	49 (62.0%)	163 (67.9%)	NS
HBeAg positive (n, %)	55 (69.6%)	176 (73.3%)	NS
PTA (%)	96.00 (73.85–117.07)	100.00 (75.00–129.73)	0.016
PLT (×10^9^/L)	164 (121–218)	192 (133–257)	<0.001
ALT (U/L)	220 (51–275)	120 (47–277)	<0.001
AST (U/L)	156 (47–237)	75 (34–183)	<0.001
HBV DNA (log_10_copies/mL)	6.66 (4.59–8.08)	6.99 (5.15–8.14)	NS
Necroinflammation grade			
<A2	26 (32.9%)	135 (56.3%)	<0.001
≥A2	53 (67.1%)	105 (43.8%)	
Fibrosis stage			
<F2	9 (11.4%)	52 (21.7%)	0.044
≥F2	70 (88.6%)	188 (78.3%)	

Parameters are expressed as median (10–90% percentile) or number (%)

PTA, prothrombin activity; PLT, platelet; ALT, alanine aminotransaferase; AST, aspirate aminotransferase; NS, not significant

Univariate analysis of clinical features indicated that lower PLT count (P = 0.016) and higher ALT and AST (P = 0.009 and P = 0.001) were associated with significant necroinflammation, while none of the parameters we investigated were associated with significant fibrosis. Clinical parameters independently associated with significant necroinflammation in univariate analysis were included in multivariate analysis. The multivariate analysis demonstrated that lower PLT count (OR: 0.982, 95%CI: 0.968–0.996; P = 0.010) and higher AST (OR: 1.014, 95%CI: 1.001–1.026; P = 0.036) were independently associated with significant necroinflammation.

## Discussion

The Asian-Pacific region is known to be endemic for HBV. Older data from the 1990s found the highest prevalence of HDV in Pakistan (58.6%) and Iran (2–20%) [25]. More recently, a study in Taiwan determined the prevalence of HDV in HBsAg carriers to be 15.3% (56/366) [26]. In China, testing for HDV is limited and the burden of HDV is likely underestimated. The main reason for the lack of testing is the common belief that HDV is a rare condition that is unlikely to be clinically relevant. Through this large retrospective cross-sectional study, we are advancing the current understanding of HDV in China and demonstrating its clinical importance. This study on the epidemiological, clinical and histological characteristics of HBV/HDV co-infection may provide a baseline for future study of HDV in China.

The prevalence of HDV previously determined among a small sample of patients in southern China was 2.1% (6/282) [27]. The prevalence of HDV in Guangzhou has been previously reported to be 13% in 1990 [28]. Our study shows a prevalence of HDV infection of 6.5%. Comparisons between this study and our study are limited by different methods of detection and a much smaller sample size (N = 74) [28]. Overall, given our large sample size, we believe our estimate to be closer to true prevalence and propose the burden of HDV in China may in fact be much higher than previously believed. Our results also demonstrate higher prevalence of HDV co-infection in patients older than 50, and prevalence rates remained fairly stable during the time period under study (Fig. 2). These may imply a decreasing trend of HDV as it is much less prevalent in the younger patients.

Co-infection with HDV is associated with diverse patterns of reciprocal inhibition of viral replication [29]. Studies have demonstrated that HDV suppresses HBV replication with most patients being HBeAg negative and with lower HBV DNA levels in contrast to patients with HBV mono-infection [11], [14], [30], [31], our findings indicated more significant suppression was found in chronic hepatitis patients with HBV/HDV co-infection. The potential virological mechanisms of HBV suppression by HDV may be the inhibition of HBV enhancers through HDV proteins p24 and p27 [32]. Also consistent with prior studies, our HBV/HDV co-infected cohort had higher levels of ALT and AST and lower levels of PLT and PTA [16], [30].. Furthermore, HBV/HDV co-infected patients are about 1.43 times more likely to progress to ESLD than patients with HBV mono-infection. These results suggest that HBV/HDV co-infection leads to more rapidly progressive liver disease in patients with HBV mono-infection. The identification of risk factors predicting the development of ESLD is very important in the long-term management of HBV/HDV co-infected patients. Of note, none of the most frequently used clinical scores to predict the outcome of liver disease like the Model for End-stage Liver disease (MELD) or Child–Pugh scores have been evaluated in HBV/HDV co-infection. Our results demonstrate male gender and age over 50 is associated with ESLD. A recent study further confirmed our results by following 75 HBsAg-anti-HDV-positive patients with HDV for up to 16 years. It is suggested the baseline-event-anticipation (BEA) score, including variables of age older than 40 and male sex, predicts with a very high accuracy the development of liver-related complications in patients with HDV [22].

The mechanism of progression to ESLD in HBV/HDV co-infected patients has not yet been elucidated. While HDV plays an important role, the association between HBV DNA levels and clinical outcomes is ill defined. Among chronic hepatitis patients, HBV/HDV co-infected patients had lower HBV DNA levels compared to HBV mono-infected patients (P = 0.003). Conversely, among ESLD patients, those with HBV/HDV co-infection had higher HBV DNA levels (P<0.001). One prior study has also shown higher detection rates, but not levels of HBV DNA, in patients with LC and HCC than in patients with acute infection [33]. It is well known that high HBV viremia in early phase is one of the most important predictors of disease progression in CHB patients [34], [35]. Furthermore, research has shown the presence of both HBV DNA and HDV RNA at baseline is associated with a higher incidence of LC and HCC [36]; however, dynamic changes of viremia were not measured in these studies. Our study shows HDV is associated with higher HBV DNA levels in ESLD patients. These findings imply that besides HDV, rapid progression to ESLD in HBV/HDV co-infected patients may also be mediated by increasing HBV DNA levels in the late-phase.

Histological abnormalities in CHD patients were more severe than those in CHB patients matched for sex, age and HBV status, confirming previous research that CHD patients tend to progress to more severe liver disease [37]. In a previous study on CHD patients of different races, 48% were found to have significant fibrosis, and clinical parameters to predict histological changes could not be identified [30]. In another smaller study, 76% of patients had advanced fibrosis [38]. Our previous study showed lower HBV DNA levels were associated with significant necroinflammation in CHB patients [23], but this was not the case in CHD patients. Our results suggest that similar to CHB patients, AST values are much more specific than ALT values in evaluating the severity of liver injuries in CHD patients [23], [39]. Based on these results, clinicians should consider liver biopsy in CHD patients to evaluate the severity of liver disease [19], [20]. Since liver biopsy being invasive and the high frequency of significant fibrosis, CHD patients may recommend antiviral therapy without biopsy confirmation.

Several limitations exist in this study. The first is that our cohort is derived from a single urban hospital in Guangzhou and may not be representative of all patients in the Guangdong province. However, Guangzhou is the economic and healthcare center of the Guangdong province, and Guangzhou No. 8 People's Hospital has one of the largest hepatitis patient populations in Guangdong. This combined with our large sample size may still be a fairly accurate estimate of the prevalence of HDV in Guangdong. Second, almost 30% of the 11622 patients screened did not receive IgM anti-HDV testing, which may underestimate the real prevalence of HDV. This finding implies that even in hospitals specializing in liver disease, many clinicians may still neglect the importance of HDV co-infection and do not provide IgM anti-HDV testing to HBsAg positive patients. Last, the HDV RNA levels and genotypes were not measured in our study as testings are not routinely available in China. IgM anti-HDV, however, is considered a useful surrogate to determine HDV replication if molecular tests for HDV RNA are not available [21]. We also excluded patients received antiviral therapy, which is a significant population in which to consider HBV/HDV co-infection. Our concern was that the decrease and clearance of IgM anti-HDV is a predictor of spontaneous or therapy-induced disease remission and would confound our estimate of prevalence [40]. Despite these limitations, this study provides greater insight into the epidemiological, clinical and histological findings of HBV/HDV co-infection in China.

In conclusion, the prevalence of HBV/HDV co-infection in Guangdong is not low. Large-scale nationwide studies should be undertaken to better estimate disease burden within China. Our results also suggest a late-phase surge in HBV DNA may contribute to progression to ESLD in HBV/HDV co-infection. Clinicians who manage HBV infected patients need to be cognizant of HDV as a risk factor for progression to ESLD. These findings support the recommendation to consider HDV antibody screening in all patients with chronic HBV infection.
